# Antifungal *Streptomyces* spp., Plausible Partners for Brood-Caring of the Dung Beetle *Copris tripartitus*

**DOI:** 10.3390/microorganisms9091980

**Published:** 2021-09-17

**Authors:** Sung Hun Kim, Goeun Park, Jin-Soo Park, Hak Cheol Kwon

**Affiliations:** Natural Product Informatics Research Center, Korea Institute of Science and Technology (KIST), Gangneung 25451, Gangwon-do, Korea; shkim12@wooree.co.kr (S.H.K.); 092297@kist.re.kr (G.P.)

**Keywords:** dung beetle, *Copris tripartitus*, DGGE, insect-symbionts, antifungal agents

## Abstract

The dung beetle *Copris tripartitus* Waterhouse (Coleoptera: Scarabaeidae) is a coprophagous insect that lives in and feeds primarily on the feces of mammalian herbivores and is known to protect their offspring from the pathogen-rich environment by performing parental care for brood balls. Brood balls under continuous management by dung beetle are rarely contaminated by entomopathogenic fungi compared to abandoned brood balls. On the supposition that dung beetles may benefit from mutualistic bacteria that protect their offspring against fungal pathogens, we evaluated the antifungal activities of bacteria isolated from the dung beetle and brood ball. As a result, bacterial isolates, mainly streptomycetes, manifested potent and broad-spectrum antifungal activity against various fungi, including entomopathogens. Of the isolates, *Streptomyces* sp. AT67 exhibited pronounced antifungal activities. Culture-dependent and independent approaches show that this strain has occurred continuously in dung beetles that were collected over three years. Moreover, metabolic profiling and chemical investigation demonstrated that the strain produced an antifungal polyene macrocyclic lactam, sceliphrolactam, as a major product. Our findings imply that specific symbiotic bacteria of *C. tripartitus* are likely to contribute brood ball hygiene by inhibiting fungal parasites in the environment.

## 1. Introduction

Dung beetles are environmentally important for the recycling of organic matter in ecosystems by the decomposition of feces, but they are threatened by the widespread use of agrochemicals and habitat destruction due to changes in agriculture and urbanization. The dung beetle *Copris tripartitus* Waterhouse (Coleoptera: Scarabaeidae) was registered in the list of endangered insects in Korea and is only found in certain areas, such as Jeju island, where pastures are abundant for stock-farming [1,2]. The species is paracoprid and builds a nest in the soil underneath the dung pat and brings dung for feeding and breeding into the nest [2,3]. The adult dung beetle puts considerable effort into constructing brood balls for oviposition from the collected dung pieces because the environment surrounding them is unfavorable for survival and maintenance of broods [4]. In addition, the adult females stay with the brood balls until the successful emergence of the offspring [5]. On the other hand, orphaned broods who do not receive continuous care are vulnerable to parasite invasions, which eventually lead to the destruction of the egg or larva inside the brood ball [6,7]. Many studies have attempted to understand a way for the dung beetle to protect offspring via brood care, but an obvious host defense mechanism remains to be elucidated [8,9].

Insects have protected themselves and their offspring against pathogens and parasites by adopting various strategies because antagonists could significantly affect their survival and development [10]. Among the strategies, host-bacterial mutualism is effective and feasible, and is frequently found in various insects, such as ants, wasps, bees, and beetles [11,12,13,14]. The symbiotic bacteria, mainly actinomycetes, engage in the protection of their host or their food resources by means of antimicrobial substances [13,15,16,17]. As symbionts, they are often associated with hygiene improvement by inhibiting competing microorganisms. In this regard, we hypothesized that the symbiotic bacteria of *C. tripartitus* may play a role as a defensive agent for the survival of offspring in brood balls against parasites and pathogens. Of the many diverse symbionts, microbiome members in the digestive system were chosen because preparation of brood balls by dung beetles is accompanied by physiological processes, including chewing, regurgitation, and defecation, indicating that the bacterial flora of the brood ball could be closely associated with that of the beetle’s digestive system. There are already some clues supporting our hypothesis. First, the dung beetle *Onthophagus taurus* (Coleoptera: Scarabaeidae) can vertically transmit their specific endosymbiotic microbiome to their offspring through the brood ball, and the unidentified matrix materials and microbes were only found in the brood ball prepared by the female in sterile rearing conditions [18]. Second, several actinomycetes have been often isolated from the dung beetle *C. tripartitus*, and they produced secondary metabolites with various chemical structures and biological activities [19,20,21,22,23].

To specify the symbiotic bacteria beneficial to the brood care of the dung beetles *C. tripartitus*, we used a dual-culture assay, screening bacterial isolates with antifungal activity from *C. tripartitus*. In addition, we examined whether the antifungal bacteria are observed continually in dung beetles through the culture-independent approach over two years. Furthermore, we identified an antifungal metabolite of the specified endosymbiont by combined secondary metabolite profiling and bioactivity-guided.

## 2. Materials and Methods

### 2.1. Sample Collection

Dung beetles were collected from pastures in Jeju Island, Korea (33°38′ N, 126°73′ E). The beetles were sampled every August from 2012 to 2014. They were caught alive using dung-baited pitfall traps without preservative [24]. Soil and cattle dung samples were also obtained from the collection site. Brood balls, each containing a larva (approximately 3rd larval instar) of dung beetles reared under laboratory conditions were provided by the National Institute of Agricultural Sciences, Republic of Korea [25].

### 2.2. Microbial Isolation and Identification

Female beetles (*n* = 3) collected in 2012 were surface sterilized with 70% ethanol for 5 min, followed by a rinse with sterile distilled water. Each was dissected to obtain the digestive tract from the whole body. The outer layer of the brood balls (*n* = 2) was carefully scraped off to obtain a portion between the outer surface and the egg chamber. The prepared samples were then ground and suspended in saline solution. The sample suspensions were heat-shock treated at 55 °C for 10 min to stimulate germination of the actinomycete spores. Samples were then inoculated by serial dilution plating on four selective agar plates containing nalidixic acid (20 μg/mL), A1 agar (A1A, 10 g soluble starch, 2 g yeast extract, 4 g peptone, 18 g agar in 1 L distilled water), Actinomycete isolation agar (AIA), Czapek-Dox agar (CDA), and Potato-Dextrose agar (PDA). During incubation at 25 °C for three weeks, bacterial and fungal strains were isolated in pure culture and maintained on YME agar (YMEA, 4 g yeast extract, 10 g malt extract, 4 g dextrose, 18 g agar in 1 L distilled water) and PDA until further processing. The isolated strains were identified based on their morphological characteristics and molecular data. Genomic DNA (gDNA) of the bacterial strains was extracted using a QIAamp DNA Mini Kit (Qiagen, Hilden, Germany) and then used as a DNA template for PCR with primers 27F and 1492R to amplify the partial 16S rRNA gene [26]. The isolation of the DNA from fungal mycelia was performed using a DNeasy Plant Mini Kit (Qiagen). The extracted DNA was used as a template for amplification of the fungal ITS region using primers ITS1 and ITS4 [27]. The resultant PCR products were purified using a QIAquick PCR purification kit (Qiagen). The amplified DNA were ligated with pGEM-T easy vector (Promega) and transformed into *Escherichia coli* DH5α. The purified plasmids were sequenced on an ABI 3730XL DNA Sequencer (Applied Biosystems, Foster City, CA). Nucleotide sequences were edited and assembled using the BioEdit program [28]. The sequences were compared using the BLASTN (https://blast.ncbi.nlm.nih.gov/Blast.cgi, accessed on 10 May 2021.) for performing similarity searches from rRNA/ITS databases consisting of 16S ribosomal RNA of bacterial and archaea type strains [29]. For phylogenetic analysis, each sequence was aligned using the ClustalW multiple sequence alignment program [30]. The phylogenetic tree was constructed using the Maximum Likelihood (ML) method applied to pairwise sequence distances calculated by the general time reversible (GTR) model with the MEGA program [31,32]. In addition, partial *rpoB* gene of antifungal isolates was obtained by PCR amplification using a primer set for *rpoB*-P2 [33] and purification, and sequenced. Additional phylogenetic tree of the concatenated sequences of 16S rRNA and *rpoB* genes was constructed using the ML method and GTR model.

### 2.3. Antifungal Screening by the Dual Culture Assay

**Figure 1:**
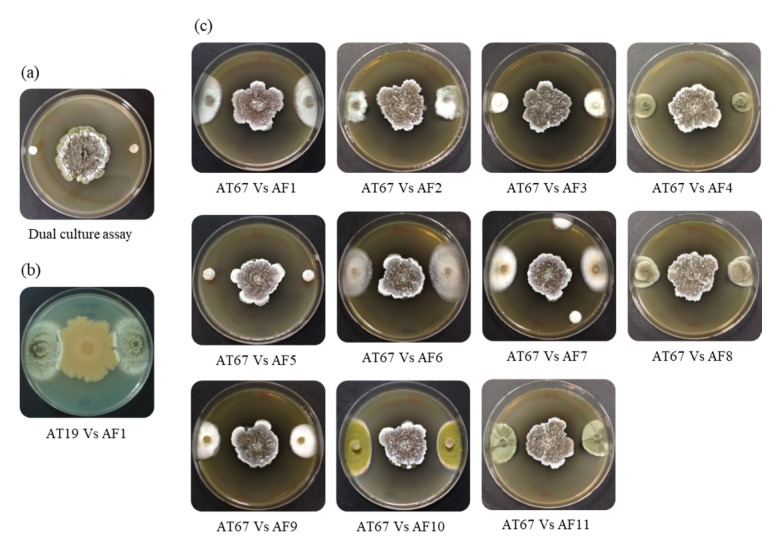
Dual culture antifungal assays with Actinobacteria isolated from dung beetles (*Copris tripartitus*) or their dung balls. (**a**) The experimental figure of the dual culture assay, (**b**) Representative result of bacterial isolate with no antifungal activity (e.g., AT19 vs. AF1), (**c**) Antifungal activity of strain AT67 against 11 fungi (AF1–AF11).

The bacterial strains isolated from dung beetles and brood balls were primarily screened for antifungal activity against ubiquitous soil fungi (AF1: *Aspergillus fumigatus* ATCC 26,430 and AF2: *Aspergillus flavus* ATCC 16883) and entomopathogenic fungi (AF3: *Beauveria bassiana* ATCC 7159 and AF4: *Metarhizium anisopliae* ATCC 20500) by the dual culture assay. The assay was conducted as follows. Each bacterial strain was cultivated in 10 mL of YME/TSB with shaking at 250 rpm for three days at 28 °C and then harvested by centrifugation. The obtained cell paste was washed twice with distilled water and re-suspended in 100 µL of fresh medium [34]. Of the concentrated cell suspension, 20 µL was inoculated onto the center of YME agar plates and incubated for three weeks at 25 °C. After incubation, two agar plugs (6 mm diameter) with growing mycelium of the test-fungi grown on PDA plates were placed on both sides 15 mm away from the resident colony (Figure 1a). After seven days, the antifungal activity was determined by measuring the distance between the edges of the fungal mycelium and the antagonistic bacterium. Subsequently, the isolates with antagonistic activity against at least one of the four tested fungi were also tested against the seven fungi isolated from the brood balls in the same manner as above. The experiment was repeated three times to confirm reproducibility.

### 2.4. Extraction of Nucleic Acids from Samples

The dung beetles (12 females and 12 males) collected in 2013 and 2014 were surface sterilized and dissected to obtain their digestive tract. Each tissue sample was homogenized in lysis buffer (20 mM Tris-Cl, 2 mM sodium EDTA, 1.2% Triton X-100 and 20 mg/mL lysozyme at a pH of 8.0) with a 5 mm stainless steel bead using a TissueLyser (Qiagen). The DNA from the homogenates was isolated using the QIAamp DNA Mini Kit (Qiagen) according to the manufacturer’s instructions. The prepared brood ball (*n* = 8) and cattle dung (*n* = 3) samples were homogenized with glass beads by vortexing and then centrifuged at 14,000 rpm for 1 min to pellet stool particles. The supernatants were used for DNA extraction with the use of the QIAamp DNA Stool Mini Kit (Qiagen). DNA extraction from the habitat soil samples (*n* = 2) was performed using the PowerMax^®^ Soil DNA Isolation Kit (MO BIO, Carlsbad, CA, USA) following the manufacturer’s protocol. The extracted DNA samples were purified using PowerClean DNA Clean-up kit (MO BIO) to eliminate PCR inhibitors. The final DNA extracts were checked for purity and concentration using a NanoDrop spectrometer ND-1000 (NanoDrop Technologies, Wilmington, DE, USA) and stored at −20 °C until further processing.

### 2.5. Nested PCR Amplification

Nested polymerase chain reaction (nested PCR) was applied for specific and sensitive amplification of the target genes in the community. The amplification of the V6/V7 region of the 16S rRNA genes from the extracted DNA samples was performed using specific primer sets for the genus *Streptomyces*, including the isolates with antifungal activity against tested fungi. The Actinomycetes-specific primers 243F and 1378R (Table S1) were used as described by Heuer et al. [35] in the first round PCR. The second PCR was performed with internal primers 984F/GC (containing a GC clamp [36]) and DBstR (Table S1) as follows: 95 °C for 3 min, followed by 25 cycles of 30 s at 95 °C, 20 s at 54 °C, and 30 s at 72 °C, with a final extension for 7 min at 72 °C. The PCR amplification yielded fragments of approximately 227 bp in size. The primer DBstR was constructed based on the alignment of the 16S rRNA gene sequences of 15 *Streptomyces* isolates and their closest phylogenetic relatives in this study. The PCR products were diluted to the same concentration (500 ng DNA/µL) using a NanoDrop spectrometer ND-1000 (NanoDrop Technologies) and used for DGGE analysis.

### 2.6. DGGE Analysis

The existence of the antifungal isolates in the samples derived from dung beetles was studied by denaturing gradient gel electrophoresis (DGGE) analysis with the DCode^TM^ Universal Mutation Detection System (Bio-Rad Laboratories, Hercules, CA, USA). For rapid identification, the DGGE marker was constructed by mixing the 16S rRNA gene fragments of the five strains (strains AT20, AT67, AT92, AT95, and AT99) amplified with primer sets 1 and 2 (Figure S1 and Table S1) and were co-electrophoresed with the samples. In the DGGE analysis, the PCR products were loaded onto 10% (*w*/*v*) polyacrylamide gels (40% acrylamide/bis solution, 37.5:1, Bio−Rad) with a linear gradient of 40–65% denaturant (100% denaturant consisted of 7 M urea and 40% (*v*/*v*) formamide). The gels were run at 60 V for 18 h in 1× TAE buffer (40 mM Tris-acetate, 1 mM EDTA, pH 8.3) kept at a constant temperature of 60 °C. After electrophoresis, the gels were stained with ethidium bromide (EtBr) for 30 min and then photographed under a UV transilluminator.

### 2.7. Identification of DGGE Bands

The DGGE bands that migrated at the same positions with respect to the DGGE marker were identified by directly sequencing the re-amplified DNA from the bands. The bands stained with SYBR Green I were excised using a sterile surgical blade on UltraBright LED Transilluminator (Maestrogen, Las Vegas, NV, USA). The excised bands were homogenized using a disposable homogenizer and then purified using the QIAquick gel extraction kit (Qiagen). The purified DNA samples were re-amplified by PCR with the primers 984F and DBstR (Table S1) and were sequenced.

### 2.8. HPLC analysis of Culture Extracts

The 16 antifungal strains were subjected to chemical analysis to profile their secondary metabolites. Each strain was cultured in YME liquid medium with constant shaking at 28 °C for seven days. The culture broths were extracted with ethyl acetate and analyzed using an Agilent 1200 series LC/MS system (Agilent, Wilmington, USA) equipped with a Phenomenex Luna C18(2) column (150 × 4.6 mm, 5 μm). The mobile phase was eluted with a linear aqueous acetonitrile gradient from 10 to 100% in 0.05% formic acid at a flow rate of 0.7 mL/min. All samples were injected at the same concentration (1 mg/mL) in the LC/MS system. The obtained HPLC chromatograms were compared with BioNumerics software (Version 7.5; Applied-Maths, Sint-Martens-Latem, Belgium). After 16 chromatograms were processed (baseline subtraction and smoothing), they were analyzed using the Dice similarity coefficient based on the presence/absence of the peaks and clustered by UPGMA (unweighted pair-group method averages) cluster analysis [37].

### 2.9. Culture and Extraction of Strain AT67

Strain AT67 was cultured aerobically in YME liquid medium (25 mL) with shaking at 200 rpm for three days at 28 °C. The seed culture was transferred into 1 L Erlenmeyer flasks containing 500 mL of YME liquid medium with 1 percent (*v*/*v*) inoculums. The culture flasks (16 L) were incubated at 28 °C on an orbital shaker at 200 rpm for 14 days. The non-ionic macroreticular resin, Amberlite XAD-7 (20 g/L, Sigma), was employed in the extraction of organic material from the culture broth of strain AT67. The adsorbed resin was washed with deionized water and then extracted in acetone. The acetone extract was concentrated on a rotary evaporator under reduced pressure.

### 2.10. Separation and Bioassay of Bioactive Fraction

For identification of antifungal metabolites produced by strain AT67, the culture extract was subjected to SiO_2_-column chromatography (Kieselgel 60, 0.063–0.200 mm, Merck) with hexane/ethyl acetate and ethyl acetate/methanol gradients as eluents to yield five fractions, F1–F5. The antifungal activity of each fraction was determined by their minimal inhibitory concentration (MIC) values against fungal strains AF10 and AF11 closely related to *A*. *flavus* and *M*. *anisopliae*, respectively. The crude extract and fractions were diluted by serial dilutions with DMSO, ranging from 0.002 to 2 mg/mL. Amphotericin B was used as a positive control at the same concentration. The fungal spore was harvested with 0.1% Tween 80 and final spore suspensions (approximately 1 × 10^4^ spores/mL) were added to each well of a sterile 96-well microtiter plate. The fungi were incubated on Potato-Dextrose broth at 28 °C for two days and observed by the naked eye to determine the lowest concentration that inhibited fungal growth.

## 3. Results

### 3.1. Microbial Isolation from the Dung Beetle and Brood Balls

Microbial isolation from the digestive tracts of female beetles and brood balls yielded 256 bacterial isolates (119 strains from the beetle’s digestive tracts and 137 strains from the brood balls) and seven fungal isolates (from the brood balls) on types of four selective agar media. Two hundred and fifty-six isolates were again simplified to 120 strains morphologically. The isolated bacterial strains showed a high diversity, but there was no significant difference between the two bacterial groups that originated from dung beetles and brood balls in morphological and molecular data.

### 3.2. Antifungal Activities of Isolates Derived from the Dung Beetle

**Figure 2:**
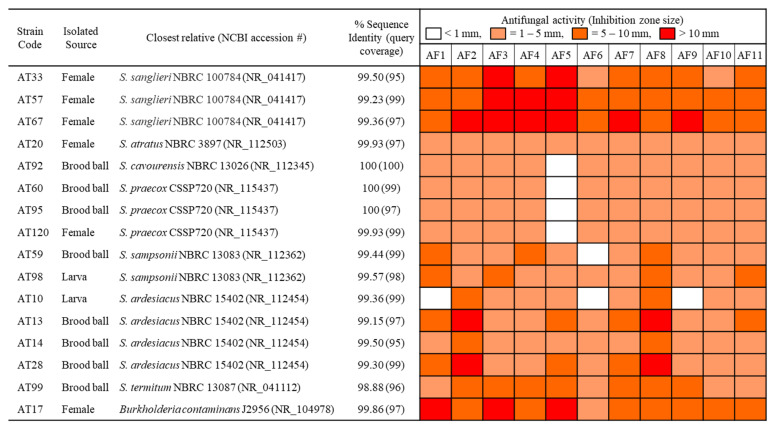
Identification and activity spectrum of antifungal bacterial strains isolated from the dung beetle, *C. tripartitus.* The bacterial isolates determined by primary antifungal screening were identified by the partial 16S rRNA gene sequences amplified using the universal primers 27F and 1492R. The antifungal activity against 11 fungi was evaluated by measuring inhibition zone size in the dual culture assays. The tested fungi were as follow: AF1: *Aspergillus fumigatus*, AF2: *A. flavus*, AF3: *Beauveria bassiana*, AF4: *Metarhizium anisopliae*, AF5: *Trametes* sp., AF6: *Fusarium* sp., AF7: *Penicillium* sp., AF8: *Cladosporium* sp., AF9: *Penicillium* sp., AF10: *Aspergillus* sp., AF11: *Metarhizium* sp.

**Figure 3:**
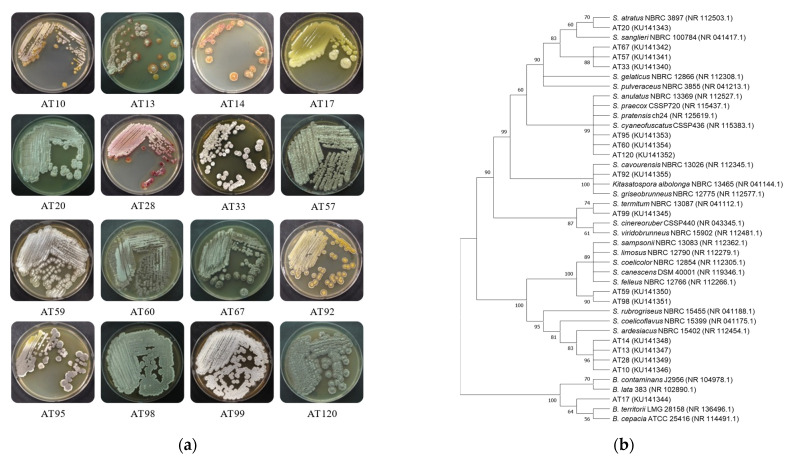
Antifungal bacterial strains isolated from dung beetles and brood balls. (**a**) Morphological characteristics of the 16 antifungal bacterial strains on YME agar plate after 20 days of cultivation. (**b**) Phylogenetic tree by Maximum Likelihood method based on the partial 16S rRNA gene of the 16 antifungal bacterial isolates and the close related members. Bootstrap values (>50%) obtained with 1000 re-samplings are given at the branch-points and branches in less than 50% are collapsed. GenBank accession numbers are indicated in parentheses. Bar, 0.02 nt substitutions per site.

Initial screening to evaluate the antifungal activity of the bacterial isolates sorted out 16 strains from 120 morphologically different strains (13% of screened isolates), showing antifungal activity against at least one of the fungal strains AF1–AF4 tested (Figure 2). Moreover, these selected sixteen isolates exhibited broad-spectrum antifungal activities (Figure 2) against additional fungal strains AF5–AF11 isolated from the brood balls. They showed clear inhibition zones against the tested fungi (Figure 1c), whereas the inactive isolates were covered by the fungal mycelium within three to five days (Figure 1b). Five strains (AT17, AT33, AT57, AT67, and AT99) were found to possess potent and broad-spectrum antifungal activities with larger inhibition zones (on average >8 mm in size) against all the tested fungi (Figure 2). Above all, strain AT67 exhibited the most pronounced antifungal activity against all the tested fungi. Except for strain AT17 belong to *Burkholderia* sp., all the antifungal strains showed typical morphological features of streptomycetes, such as an opaque rough surface, characteristic color, and non-spreading (Figure 3a).

### 3.3. Identification and Phylogenetic Analysis of Antifungal Isolates

The 16 antifungal strains were identified at the species level by 16S rRNA gene sequence analysis, and the results are presented in Figure 3. According to the comparative analysis in the GenBank database, the sequence similarities to their closest phylogenetic relative showed high values ranging from 99 to 100%. A phylogenetic tree was constructed using 16S rRNA sequences of the 16 strains and their close neighbor strains with similarity (Table S2). The tree was created by maximum likelihood method under general time reversible model with a discrete gamma distribution and invariable sites model (GTR + G + I) calculated as best model by Mega-X. The phylogenetic tree based on 16S rRNA sequences revealed that all isolates, except for AT17, were clustered with their corresponding reference strain with bootstrap resampling values higher than 50% and belonged to the genus *Streptomyces* (Figure 3b), indicating that streptomycetes are highly associated with these dung beetles. The isolates were divided into eight groups at distinct branches as follows: Strains (1) AT33, AT57, and AT67; (2) AT20; (3) AT60, AT95, and AT120; (4) AT92; (5) AT99; (6) AT59 and AT98; (7) AT10, AT13, AT14, and AT28; and (8) AT17. These obtained 16 sequences have been submitted to the NCBI GenBank database under accession numbers KU141340 to KU141355, respectively. In order to verify that the antifungal isolates are included in a specific genus, the *rpoB* gene sequence was prepared. From BLAST search with 540 bp DNA corresponding to partial *rpoB* gene, all of the relevant strains were confirmed to belong to *Streptomyces,* except for AT17, which was classified as *Bukholderia* (Table S2). AT95 showed high similarity of 99.81% with *Kitasatospora papulosa* NRRL B-16504, which was recently demonstrated as a member of the species *S. prentensis* by multi-locus sequence analysis [38]. Further phylogenetic analysis of the antifungal strains and close strains based on the concatenated sequences of 16S rRNA and *rpoB* genes also showed the analogous to Figure 3b clustering into eight groups except for independently located AT95 from AT60 and AT120 (Figure S2).

Seven fungal strains isolated from the brood balls were also identified at the genus or species level by morphological imaging and ITS sequencing data. According to BLAST searches of the GenBank database, they were identified as potential entomopathogens as follows: *Trametes* sp. (AF5), *Fusarium* sp. (AF6), *Penicillium* sp. (AF7), *Cladosporium* sp. (AF8), *Penicillium* sp. (AF9), *Aspergillus* sp. (AF10) and *Metarhizium* sp. (AF11). The fungal strains AF10 and AF11 belonged to the same genotype group as those that were used in initial screening for antifungal activity (AF2: *A. flavus* ATCC 16,883 and AF4: *M. anisopliae* ATCC 20500).

### 3.4. Association between the Dung Beetle and Antifungal Isolates

To investigate the symbiotic association of the bacterial isolates with antifungal activity in the dung beetle ecosystem, the PCR-DGGE method was tried but there was difficulty in amplifying the 16S rRNA gene of bacterial strains belonging to the genus *Streptomyces* from environmental samples because the group has a high genomic G + C content and a low abundance, which leads to poor amplification efficiency in a competitive PCR [35]. Initial DGGE analysis on the 16S V3 region amplified with universal bacterial primers (341F/GC and 518R) revealed only the presence of Enterobacteriaceae, including *Citrobacter*, *Klebsiella*, *Proteus*, and *Serratia* species in both dung beetles and brood balls (data not shown). To solve these issues, we needed to design genus-specific primer pairs for the amplification of *Streptomyces* 16S rRNA gene. The primer DBstR was constructed based on an insertion sequence of 5 or 6 nt, which is typical of *Streptomyces* 16S rRNA genes (Figure S3). It amplified the hypervariable V6/V7 region suitable for specific detection of *Streptomyces* species with a primer 984F/GC [39]. Preliminary PCR experiments using the primer pair 984F/GC and DBstR on DNA isolated from *Streptomyces* and non-*Streptomyces* strains yielded specific amplification of only *Streptomyces* strains, suggesting specificity in DNA amplification (Figure S3).

**Figure 4:**
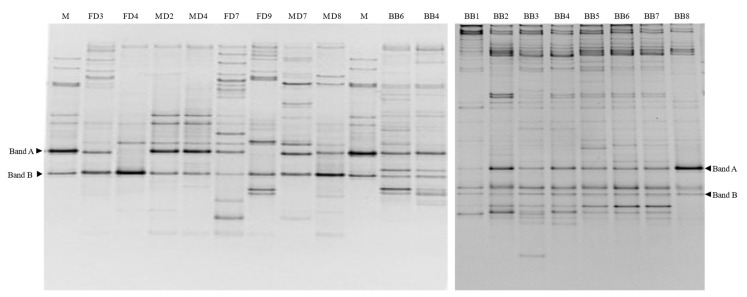
Representative DGGE for detection of Streptomyces antifungal isolates in samples derived from the dung beetles (**left**) and brood balls (**right**). (FD3, FD4) Digestive tracts of female dung beetles collected in 2013; (MD2, MD4) Digestive tracts of male dung beetles collected in 2013; (FD7, FD9) Digestive tracts of female dung beetles collected in 2014; (MD7, MD8) Digestive tracts of male dung beetles collected in 2014; (BB1-BB8) Brood ball; M: Marker (Band A: AT92, AT95 and AT99, Band B: AT20 and AT67]).

Though not covering all bacterial species, the DGGE profiles, which were generated with the genus-specific primer sets, consisted of abundant bands, implying that the dung beetle possessed a microbiota of multifarious bacteria related to streptomycetes. From the overall pattern of the DGGE bands, it was difficult to clearly recognize the distinguishable characteristic in the endosymbiotic bacterial community of the beetle’s digestive tracts because each individual beetle showed a quite different pattern (Figure 4 and Figure S4). In contrast, the DGGE profiles in brood balls exhibited a regular banding pattern, including several predominant bands (Figure 4). Prior to analysis, a DGGE marker was prepared including antifungal strains, AT67, AT92, AT99, AT20, and AT 95 (Figure S1). At the marker, the strains were appeared divided into two bands (band A: AT92, AT99, and AT95, band B: AT67 and AT20). In the DGGE profiles, band A and B were observed in every brood ball and some dung beetles (Figure 4 and Figure S4).

According to these DGGE results, there is no distinct gender difference regarding the endosymbiotic bacteria corresponding to band B (Figure S4). In comparison to the DGGE profiles in the beetle’s digestive tract, band B was weakly present at almost the same intensity in all brood balls (Figure 4). In addition, band B was not present in the habitat soil and cattle dung samples (Figure 2). Additionally, band A appeared in cattle dung and not in the habitat soil. Re-amplification and sequencing of each band corresponding to the band B position revealed that they had 100% sequence similarity with the V6/V7 region in the 16S rRNA of *S. sanglieri* (NR_041417), which is the closest relative of strain AT67. Each band corresponding to the band A position was also identified with 100% sequence homology to *S. termitum* (NR_041112), which is the closest relative of strain AT99.

### 3.5. Chemical Profile of 16 Antifungal Strains

**Figure 5:**
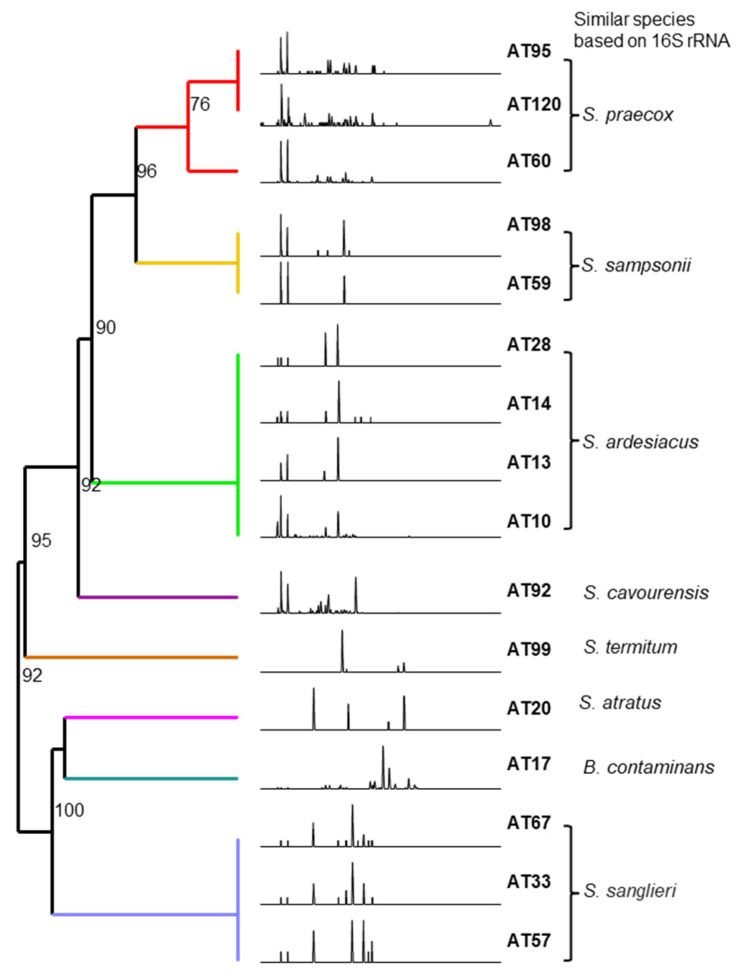
Similarity dendrogram constructed based on HPLC chromatograms with the Dice coefficient and UPGMA method using BioNumerics software.

HPLC chromatograms of culture extracts from the 16 antifungal strains are summarized in Figure 5. The comparative metabolic analysis revealed that the strains that clustered in the same phylogenetic branch showed a similar metabolic profile with a slight difference in the relative peak intensities (Figure 5). For instance, strains AT33, AT57, and AT67 clustered together in a branch presented an almost similar metabolic pattern, but the intensity of each peak was slightly different, while *Burkholderia* sp. AT17 in the discrete branch exhibited a completely different metabolic pattern. Similarity analysis of the HPLC chromatograms with BioNumerics software revealed that strains belonging to the almost identical species level were clustered together with a high similarity percentage and exhibited similar clustering to those of the phylogenetic tree analysis based on their 16S rRNA sequences (Figure 3b and Figure 5). This implies that the secondary metabolites profile might be also useful for bacterial chemotaxonomic classification.

### 3.6. Antifungal Metabolite Produced by Strain AT67

**Figure 6:**
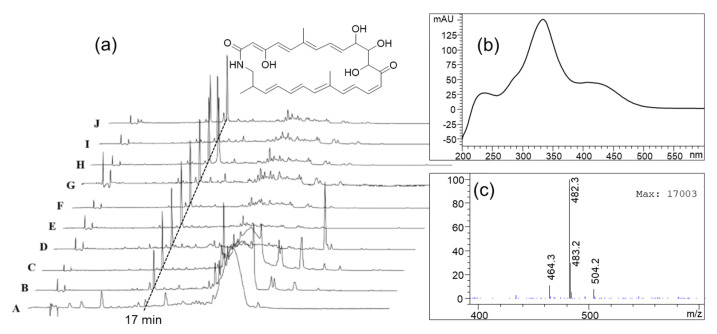
Antifungal compound, sceliphrolactam produced by AT67. (**a**) HPLC chromatograms derived from extracts of culture and inhibition zone (IZ) on agar plates of dual culture between AT67 and fungal strains (A: broth culture of AT67, B: Agar culture of AT67, C: IZ between AT67 and AF1, D: IZ between AT67 and AF2, E: IZ between AT67 and AF5, F: IZ between AT67 and AF6, G: IZ between AT67 and AF7, H: IZ between AT67 and AF9, I: IZ between AT67 and AF10, J: IZ between AT67 and AF11.). UV absorption (**b**) and mass spectrum (**c**) of the peak at a retention time of 17 min (UV maxima at 333 and 420 nm, *m/z* 482.3 for [M + H]^+^ and *m*/*z* 504.2 for [M + Na]^+^), indicating sceliprolactam.

Further LC-MS analysis was executed to identify the actual antifungal metabolites produced from the most potent antifungal strain AT67 and revealed the presence of a major component in the culture extract (Figure 6). The component was thought to be a polyene macrocyclic lactam through pre-screening using our in-house HPLC-MS-UV database and identified as sceliphrolactam by the comparison with previously reported HPLC and MS data (Figure 6) [40,41]. In the MIC assay, the crude extract and the three fractions (Fr3, Fr4, and Fr5) derived from AT67 showed inhibitory activity against the fungal strains AF10 and AF11 with MIC values of 0.125–0.5 mg/mL. The active fractions showed a slight difference in activity level according to the content of sceliphrolactam in the fractions. Additionally, the compound was detected in all of the inhibitory zones formed between strain AT67 and the fungi tested in dual culture assay (Figure 6). Therefore, sceliphrolactam was presumed to be a putative candidate produced from strain AT67 as an active compound against pathogenic fungi.

## 4. Discussion

Dung beetles spend most of their life close to a fecal-rich environment. Due to this unique lifestyle, they are threatened by antagonists such as entomopathogenic fungi isolated abundantly from dung balls. These fungi invade the nutrient-rich hemolymph of the insect host by using a combination of cuticle-degrading enzymes and mechanical force, eventually causing the death of the host [42,43]. Moreover, these entomopathogens can easily penetrate the insect eggs and larvae [44,45]. Thus, the beetles need a counterplan to care for their offspring. According to previous reports, e.g., when the dung beetle *C. tripartitus* is invaded by microbial pathogens, it defends itself by using an antimicrobial peptide Coprisin as a defensive immune response [46,47]. In this study, we postulated an antifungal bacterial symbiont as a defensive agent against fungal invasion in the brood care of the dung beetle *C. tripartitus* and tried to demonstrate this through culture-dependent and independent approaches.

Brood care by the dung beetle causes frequent and consistent interaction via oral secretion from the digestive tract, between the beetle and brood ball; we, thus, focused on the antifungal bacterial association derived from microbiota in dung beetles and brood balls. In this study, we revealed that most of the bacterial strains showing remarkable antifungal activity belong to *Streptomyces* spp., which are often referred to as protective symbionts in the insect–bacteria symbiosis [16,40]. The consistent presence of antifungal streptomycetes in the dung beetles *C. tripartitus* was evaluated by culture independent method using PCR-DGGE with primer set designed for *Streptomyces* 16S rRNA genes. This allowed us to assess the relative proportions and distribution of antifungal *Streptomyces* isolates through a semi-quantitative analysis based on the DGGE bands and showed that *Streptomyces* sp. AT67 and AT99 were common and major species in the brood balls and digestive tract of *C. tripartitus*. However, DGGE results for the habitat soil or cattle dung suggested a different origination of the two species. The AT67 strain did not occur in the habitat soil and cattle dung, which are the main materials for the construction of brood balls, whereas the AT99 strain was present as a major species in the cattle dung, indicating that the AT99 strain is an external organism present in the dung beetle’s digestive system introduced by feeding. On the other hand, the presence of strain AT67 in only the brood balls and digestive tract of the dung beetles, but not in the soil or cattle dung, suggests that it may be an endosymbiont of the dung beetles. In addition, the successive occurrences of strain AT67 over three years suggest that *C. tripartitus* vertically transmits the endosymbiotic bacterium to their offspring via brood balls, which are the sole food source for the developing offspring. Recently, vertical transmission of microbiota via the food carcass has been shown in burying beetles [48,49,50]. Similarly, *C. tripartitus* may utilize the endosymbionts stored in its digestive system during the construction and maternal care of their brood balls.

Along with the plausible symbiotic association, mainly with streptomycetes, we ascertained that the potent antifungal strain AT67 produced a diffusible antifungal compound, sceliphrolactam, as a major secondary metabolite. The compound was first isolated from wasp-associated *Streptomyces* sp. and reported to possess antifungal activity against amphotericin B–resistant *Candida albicans* (MIC = 4 μg/mL, 8.3 μM). Sceliphrolactam is a polyene macrocyclic lactam and its structure is extremely fragile and labile [41]. Therefore, exposures of the compound to the environments with changeable conditions often lead to its degradation, which may consequently lead to loss of its antifungal activity [51]. Considering orphaned broods without continuous brood ball care are unsuccessful in surviving due to the parasite and pathogen invasion, there may be a link between continuous brood ball care and the antifungal compound production by streptomyces sp.

On the other hands, fungi are mainly described as entomopathogen, but they often involves a mutualistic players in insect–microbe symbiosis such as *Fusarium solani*, which is cultivated by a species of ambrosia beetle *Xylosandrus compactus* as nutrition source [52], and *Leucoagaricus gongylophorus*, which provides food to the ant *Acromyrmex octospinosus* [53]. In addition, one clade of ambrosia beetles has evolved with a mutualist of the polypore fungus *Flavodon ambrosius* facilitating wood decay by lignin-modifying enzymes [54]. Therefore, we need to investigate more systemically the fungi isolated from the brood balls to reveal their roles and functions in the insect ecosystem by further study.

To sum up these results, the dung beetle *C. tripartitus* harbors antifungal bacteria, which are mainly comprised of *Streptomyces* bacteria that are highly likely to protect its offspring in brood balls from diverse fungal pathogens. Furthermore, as antifungal symbionts, the *Streptomyces* sp. AT67 was proposed to play a significant role as a defensive agent in dung beetle-bacteria symbiosis. These defensive associations may be maintained through successful transmission to the brood ball and production of antifungal substances as a major secondary metabolite.

## Data Availability

Not applicable.

## Supplementary Materials

The following are available online at https://www.mdpi.com/article/10.3390/microorganisms9091980/s1, Figure S1: DGGE marker (M) used in this study for the rapid identification of *Streptomyces* antifungal isolates (upper) and DGGE for the detection of *Streptomyces* antifungal isolates from the habitat soil and cattle dung., Figure S2: Phylogenetic tree based on the concatenated sequences of 16S rRNA and *rpoB* genes., Figure S3: PCR result using genus-specific primer sets, Figure S4: DGGE for detection of *Streptomyces* antifungal isolates from the digestive tracts of the dung beetles collected in 2013 and 2014., Table S1: Primer sets used for PCR-DGGE, Table S2: BLASTN search results with 16S rRNA and *rpoB* sequences.
