# Supplementary material for: Antifungal Streptomyces spp., Plausible Partners for Brood-Caring of the Dung Beetle Copris tripartitus
Source: Microorganisms. 2021 Sep 17;9(9):1980. doi: 10.3390/microorganisms9091980 (PMC8466457; doi:10.3390/microorganisms9091980)
Supplement: Supplementary file 1 [file microorganisms-09-01980-s001.zip › microorganisms-1283559-supplementary.pdf]

Supplementary data

Antifungal *Streptomyces* spp., plausible partners for brood-caring of the dung beetle *Copris tripartitus*

Sung Hun Kim, Goeun Park, Jin-Soo Park* and Hak Cheol Kwon*

Natural Product Informatics Research Center, Korea Institute of Science and Technology (KIST), Gangneung, Gangwon-do, 25451, Republic of Korea

* Correspondence: jinsoopark@kist.re.kr (J.-S. Park), and hkwon@kist.re.kr (H. Kwon)

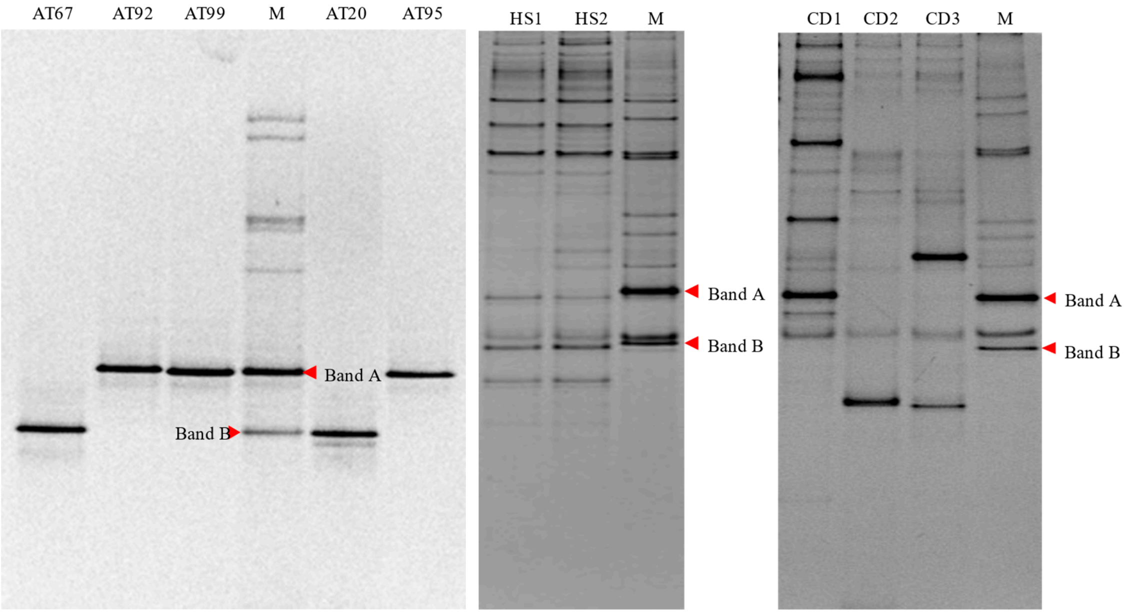

Figure S1. DGGE marker (M) used in this study for the rapid identification of *S.* antifungal isolates (left) and DGGE for the detection of *S.* antifungal isolates from the habitat soil and cattle dung (right). HS: Habitat soil, CD: Cattle dung, M: Marker [Band A: AT92, AT95 and AT99, Band B: AT20 and AT67].

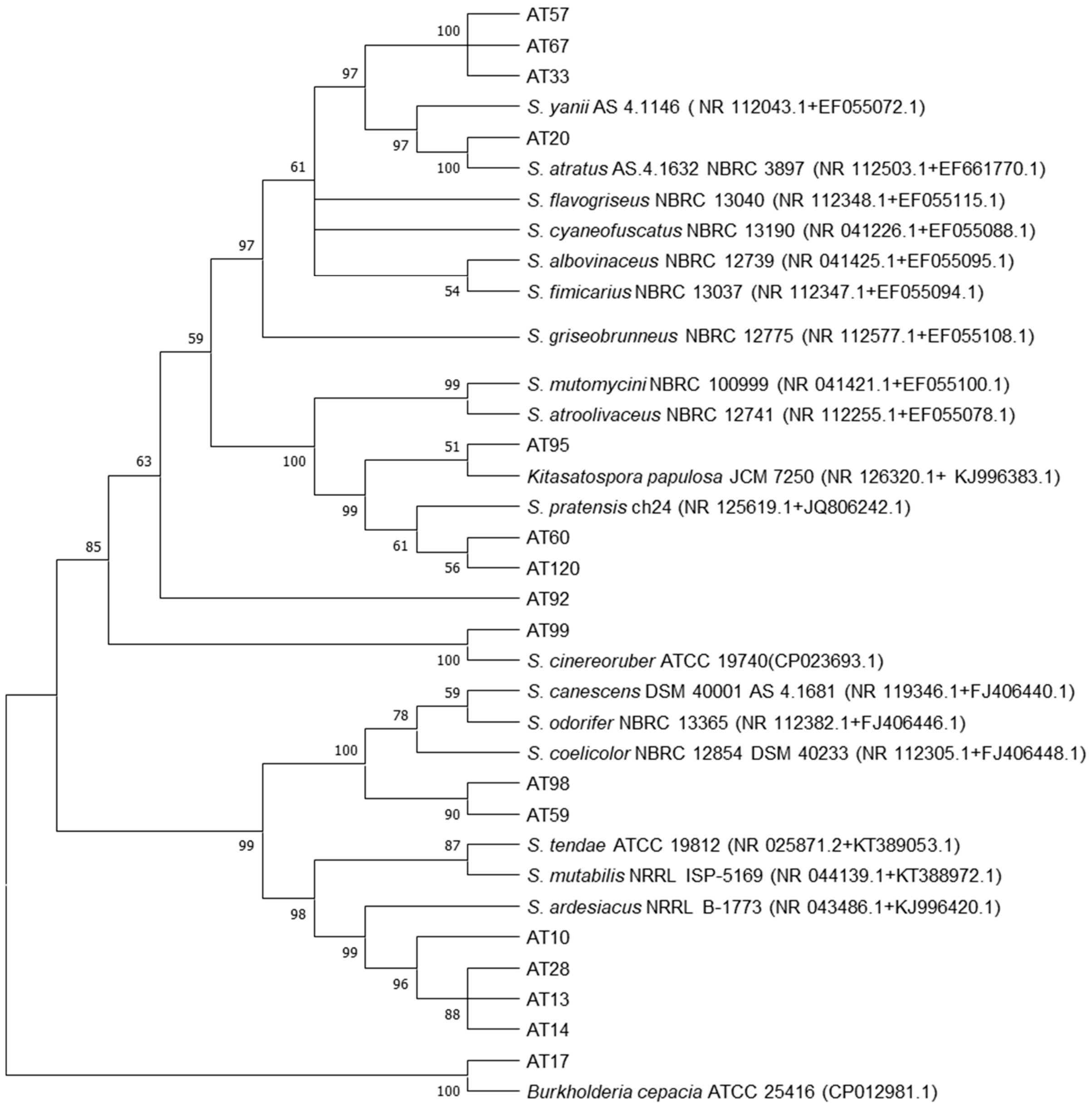

Figure S2. Phylogenetic tree based on the concatenated sequences of 16S rRNA and *rpoB* genes. The tree was constructed using the Maximum Likelihood method and General Time Reversible model. The percentage of replicate trees in which the associated taxa clustered together in the bootstrap test (1000 replicates) are shown next to the branches and branches in less than 50% bootstrap replicates are collapsed.

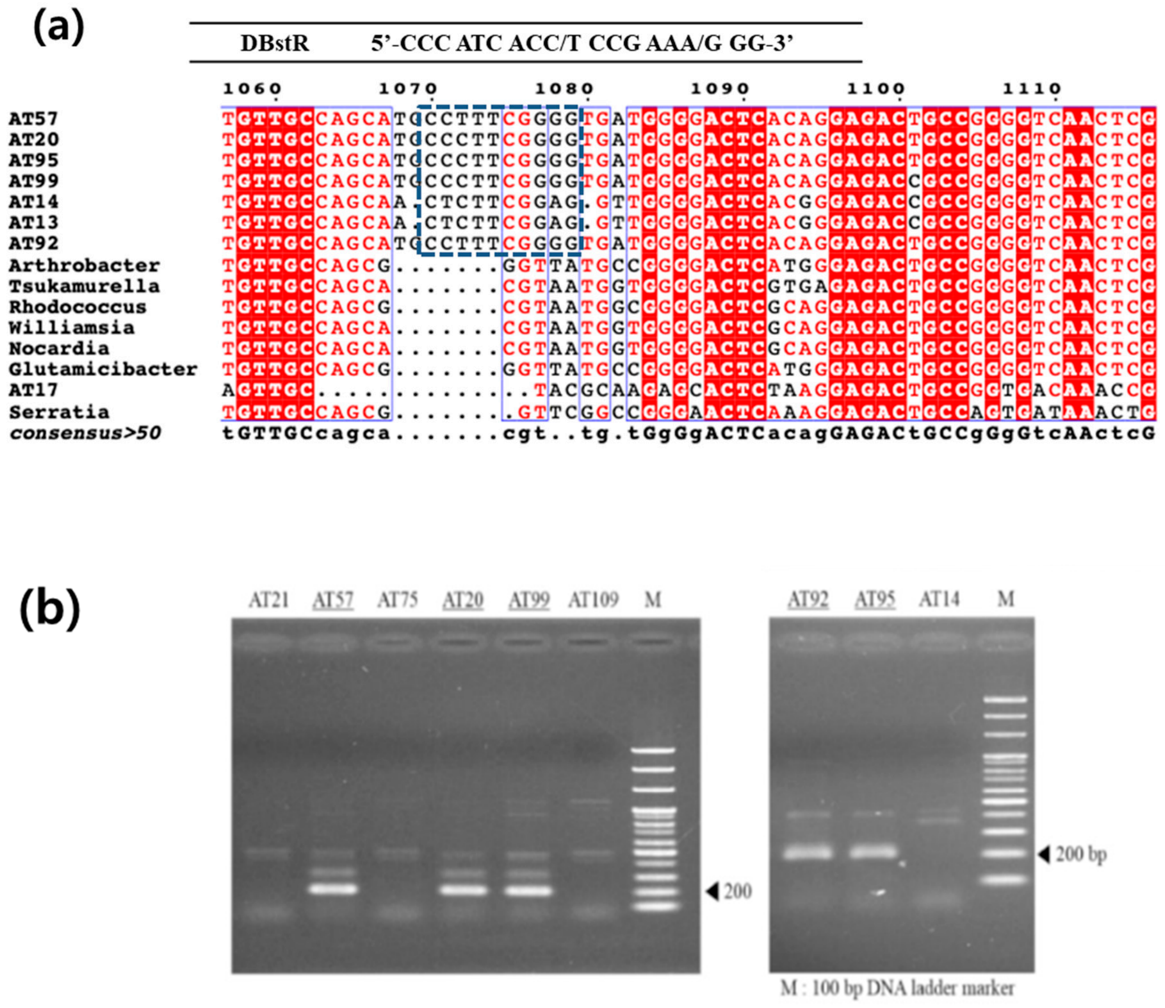

Figure S3. Design of the primer DBstR for specific ampiification of antifungal strains (a) and PCR result using the primer set (984F and DBstR) (b)

Arthrobacter agilis (NR_170400), *Glutamicibacter creatinolyticus* (NR_036769), *Tsukamurella tyrosinosolvens* (NR_042801), *Nocardia mikamii* (NR_116334), *Rhodococcus hoagii* (NR_041910), *Williamsia muralis* (NR_037083), *Serratia marcescens* (NR_036886)

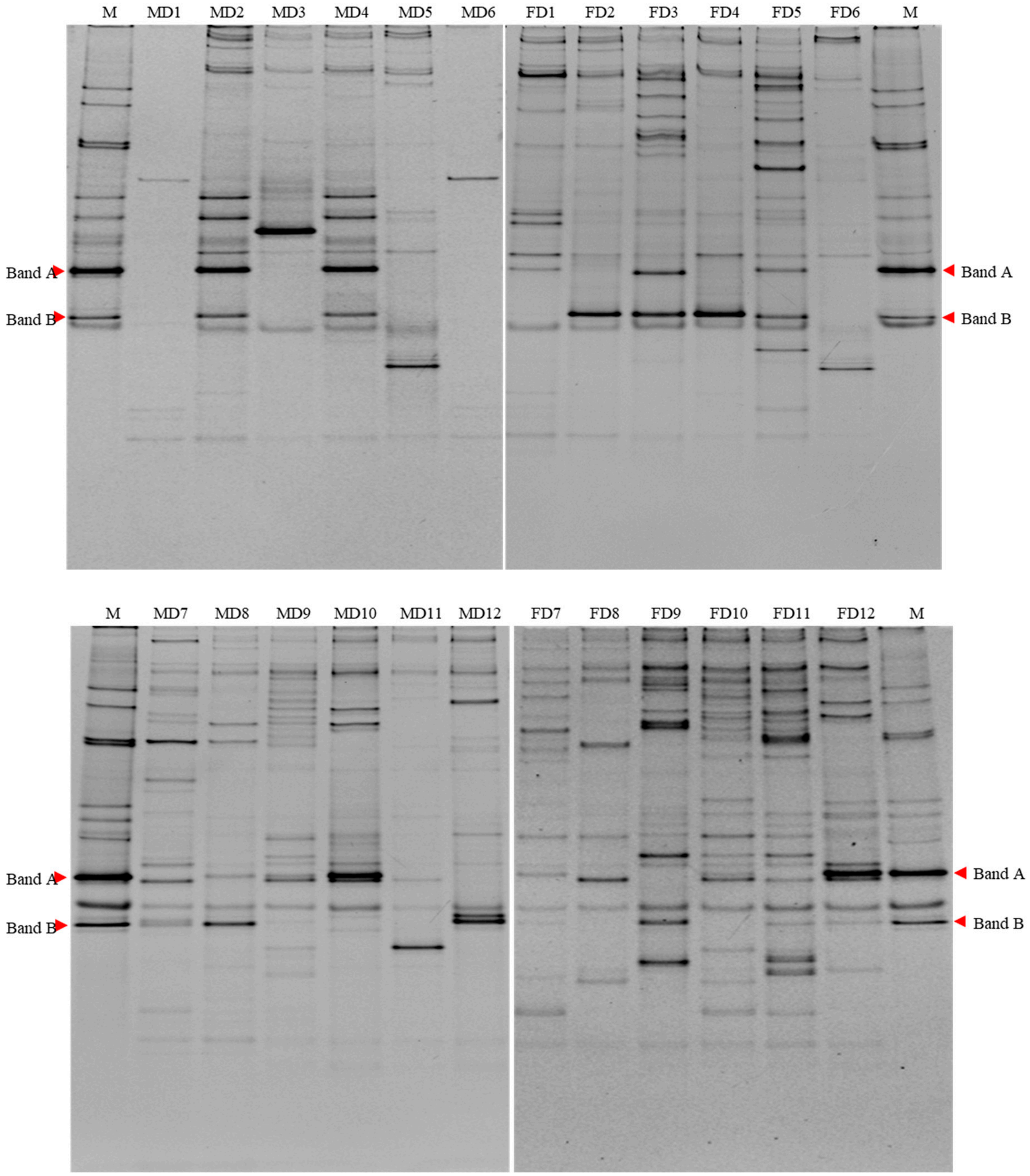

Figure S4. DGGE for detection of *S.* antifungal isolates from the digestive tracts of the dung beetles collected in 2013 (upper) and 2014 (bottom).

MD: Male dung beetle, FD: Female dung beetle, M: Marker [Band A: AT92, AT95 and AT99, Band B: AT20 and AT67].

Table S1 Primer sets used for PCR-DGGE

Primer Set	Target group	Primer name	Sequence (5´ to 3´)	Reference
**1**	Actinomycetes	243F	GGA TGA GCC CGC GGC CTA	[1]
		1378R	CGG TGT GTA CAA GGC CCG GGA ACG	[1]
**2**	S.	984F	AAC GCG AAG AAC CTT AC	[1]
		DBstR[a]	CCC ATC ACC/T CCG AAA/G GG	This study

[a] GC clamp : CGC CCG GGG CGC GCC CCG GGC GGG GCG GGG GCA CGG GGG G

* GC clamp attached at 5´ end of primer to prevent complete strand dissociation during electrophoresis [2].

Table S2 BLASTN search results with 16S rRNA and rpoB gene sequences

Strain	16S rRNA gene				rpoB gene				16S rRNA + rpoB			
	Description	Coverage	Identity (%)	Accession	Description	Coverage	Identity (%)	Accession	Description	Coverage	Identity (%)	Accession
AT10	S. ardesiacus NBRC 15402	0.99	99.36	NR_112454.1	S. aureoversilis NRRL B-3325	1	99.44	KJ996724.1	S. ambofaciens ATCC 23877	0.99	98.37	CP012382.1
	S. ardesiacus NRRL B-1773	0.99	99.29	NR_043486.1	S. tendae NRRL B-2313	1	98.52	KT389053.1	S. koyangensis VK-A60T	0.99	97.87	CP031742.1
	S. coelicoflavus NBRC 15399	0.99	99.22	NR_041175.1	S. ardesiacus NRRL B-1773	1	98.52	KJ996420.1	S. coeruleorubidus ATCC 13740	0.99	97.8	CP023694.1
	S. coelicoflavus CSSP410	0.99	99.28	NR_115371.1	S. plicatus NRRL 2428	1	98.33	KJ996182.1				
	S. rubrogriseus NBRC 15455	0.99	98.72	NR_041188.1	S. rochei NRRL 3533	1	98.15	KJ996201.1				
AT13	S. ardesiacus NBRC 15402	0.97	99.15	NR_112454.1	S. aureoversilis NRRL B-3325	1	99.81	KJ996724.1	S. ambofaciens ATCC 23877	0.98	98.17	CP012382.1
	S. ardesiacus NRRL B-1773	0.97	99.08	NR_043486.1	S. plicatus NRRL 2428	1	98.7	KJ996182.1	S. koyangensis VK-A60T	0.98	97.68	CP031742.1
	S. coelicoflavus NBRC 15399	0.97	99.01	NR_041175.1	S. tendae NRRL B-2313	1	98.52	KT389053.1	S. coeruleorubidus ATCC 13740	0.98	97.61	CP023694.1
	S. coelicoflavus CSSP410	0.96	99.36	NR_115371.1	S. rochei NRRL 3533	1	98.52	KJ996201.1				
	S. rubrogriseus NBRC 15455	0.97	98.52	NR_041188.1	S. mutabilis NRRL ISP-5169	1	98.33	KT388972.1				
AT14	S. ardesiacus NBRC 15402	0.95	99.5	NR_112454.1	S. aureoversilis NRRL B-3325	1	99.81	KJ996724.1	S. ambofaciens ATCC 23877	0.96	98.5	CP012382.1
	S. ardesiacus NRRL B-1773	0.95	99.43	NR_043486.1	S. plicatus NRRL 2428	1	98.7	KJ996182.1	S. koyangensis VK-A60T	0.96	98	CP031742.1
	S. coelicoflavus NBRC 15399	0.95	99.36	NR_041175.1	S. tendae NRRL B-2313	1	98.52	KT389053.1	S. coeruleorubidus ATCC 13740	0.96	97.94	CP023694.1
	S. coelicoflavus CSSP410	0.94	99.43	NR_115371.1	S. rochei NRRL 3533	1	98.52	KJ996201.1				
	S. rubrogriseus NBRC 15455	0.95	98.79	NR_041188.1	S. mutabilis NRRL ISP-5169	1	98.33	KT388972.1				
	S. mutabilis NRRL ISP-5169	0.97	97.54	NR_044139.1								
	S. tendae ATCC 19812	0.97	98.17	NR_025871.2								
AT17	B. contaminans J2956	0.97	99.86	NR_104978.1	B. cepacia ATCC 25416	1	100	CP034553.1	B. cepacia ATCC 25416	0.98	100	CP012981.1
	B. territorii LMG 28158	0.97	99.71	NR_136496.1	B. cepacia LMG 1222	0.98	100	EU024270.1	B. lata 383	0.98	99.86	CP000151.1
	B. cepacia ATCC 25416	0.97	99.79	NR_114491.1	B. ambifaria AMMD	1	97.96	CP009798.1	B. ambifaria AMMD	0.98	99.71	CP000440.1
	B. cepacia NBRC 14074	0.97	99.71	NR_113645.1	B. pyrrocinia DSM 10685	1	97.78	CP011503.1	B. vietnamiensis LMG 10929	0.98	99.57	CP009631.1
	B. lata 383	0.97	99.71	NR_102890.1	B. acidophila ATCC 31433	1	97.22	CP020737.1	B. stabilis ATCC BAA-67	0.98	99.5	CP016442.1
AT20	S. atratus NBRC 3897	0.97	99.93	NR_112503.1	S. atratus AS.4.1632	1	99.63	EF661770.1	S. californicus FDAARGOS_1209	0.98	99.06	CP070242.1
	S. atratus NRRL B-16927	0.97	99.93	NR_043490.1	S. yanii AS 4.1146	1	97.41	EF055072.1	S. fulvissimus DSM 40593	0.98	98.99	CP005080.1
	S. sanglieri NBRC 100784	0.97	99.86	NR_041417.1	S. flavogriseus AS 4.1884	1	96.67	EF055115.1	S. nitrosporeus ATCC 12769	0.98	98.85	CP023702.1
	S. pulveraceus NBRC 3855	0.97	99.64	NR_041213.1	S. albovinaceus AS 4.1631	1	96.67	EF055095.1	S. lunaelactis MM109	0.98	98.7	CP026304.1
	S. gelaticus NBRC 12866	0.97	99.5	NR_112308.1	S. fimicarius AS 4.1629	1	96.67	EF055094.1				
	S. yanii AS 4.1146	0.97	98.49	NR_112043.1								
	S. albovinaceus CSSP418	0.97	99.21	NR_115373.1								
	S. albovinaceus NBRC 12739	0.97	99.14	NR_041425.1								
	S. fimicarius NBRC 13037	0.97	98.99	NR_112347.1								
AT28	S. ardesiacus NBRC 15402	0.99	99.3	NR_112454.1	S. aureoversilis NRRL B-3325	1	99.81	KJ996724.1	S. ambofaciens ATCC 23877	0.99	98.18	CP012382.1
	S. ardesiacus NRRL B-1773	0.99	99.23	NR_043486.1	S. plicatus NRRL 2428	1	98.7	KJ996182.1	S. koyangensis VK-A60T	0.99	97.82	CP031742.1
	S. coelicoflavus NBRC 15399	0.99	99.16	NR_041175.1	S. tendae NRRL B-2313	1	98.52	KT389053.1	S. coeruleorubidus ATCC 13740	0.99	97.63	CP023694.1
	S. coelicoflavus CSSP410	0.98	99.29	NR_115371.1	S. rochei NRRL 3533	1	98.52	KJ996201.1				
	S. rubrogriseus NBRC 15455	0.98	98.66	NR_041188.1	S. mutabilis NRRL ISP-5169	1	98.33	KT388972.1				
AT33	S. sanglieri NBRC 100784	0.95	99.5	NR_041417.1	S. atratus AS.4.1632	1	95.93	EF661770.1	S. nitrosporeus ATCC 12769	0.96	98.93	CP023702.1

	S. atratus NBRC 3897	0.95	99.43	NR_112503.1	S. cyaneofuscatus AS 4.1612	1	95.93	EF055088.1	S. californicus FDAARGOS_1209	0.96	98.86	CP070242.1
	S. atratus NRRL B-16927	0.95	99.43	NR_043490.1	S. bacillaris ATCC 15855	1	95.74	CP029378.1	S. fulvissimus DSM 40593	0.96	98.79	CP005080.1
	S. pulveraceus NBRC 3855	0.95	99.29	NR_041213.1	S. flavogriseus AS 4.1884	1	95.74	EF055115.1	S. bacillaris ATCC 15855	0.96	98.72	CP029378.1
	S. gelaticus NBRC 12866	0.95	99.14	NR_112308.1	S. griseobrunneus AS 4.1838	1	95.74	EF055108.1	S. lunaelactis MM109 MM109	0.96	98.64	CP026304.1
	S. cyaneofuscatus CSSP436	0.95	98.86	NR_115383.1								
	S. cyaneofuscatus NBRC 13190	0.95	98.79	NR_041226.1								
	S. bacillaris NBRC 13487	0.95	98.72	NR_041146.1								
	S. griseobrunneus NBRC 12775	0.95	98.58	NR_112577.1								
	S. flavogriseus NBRC 13040	0.95	99.07	NR_112348.1								
AT57	S. sanglieri NBRC 100784	0.99	99.23	NR_041417.1	S. atratus AS.4.1632	1	95.93	EF661770.1	S. nitrosporeus ATCC 12769	0.99	98.66	CP023702.1
	S. atratus NBRC 3897	0.99	99.16	NR_112503.1	S. cyaneofuscatus AS 4.1612	1	95.93	EF055088.1	S. californicus FDAARGOS_1209	0.99	98.59	CP070242.1
	S. atratus NRRL B-16927	0.99	99.16	NR_043490.1	S. bacillaris ATCC 15855	1	95.74	CP029378.1	S. fulvissimus DSM 40593	0.99	98.52	CP005080.1
	S. pulveraceus NBRC 3855	0.99	99.02	NR_041213.1	S. flavogriseus AS 4.1884	1	95.74	EF055115.1	S. bacillaris ATCC 15855	0.99	98.46	CP029378.1
	S. gelaticus NBRC 12866	0.99	98.87	NR_112308.1	S. griseobrunneus AS 4.1838	1	95.74	EF055108.1	S. lunaelactis MM109	0.99		CP026304.1
AT59	S. sampsonii NBRC 13083	0.99	99.44	NR_112362.1	S. canescens AS 4.1681	1	100	FJ406440.1	S. koyangensis VK-A60T	0.99	99.15	CP031742.1
	S. coelicolor NBRC 12854	0.99	99.44	NR_112305.1	S. odorifer NBRC 13365	1	99.81	FJ406446.1	S. cadmiisoli ZFG47	0.99	98.31	CP030073.1
	S. limosus NBRC 12790	0.99	99.44	NR_112279.1	S. argenteolus AS 4.1693	1	99.81	EF055099.1	S. pluripotens MUSC 135	0.99	98.1	CP021080.1
	S. felleus NBRC 12766	0.99	99.44	NR_112266.1	S. vinaceus AS 4.1305	1	99.81	EF055073.1	S. tuirus JCM 4255	0.99	97.96	AP023439.1
	S. canescens DSM 40001	0.99	99.37	NR_119346.1	S. coelicolor DSM 40233	1	99.63	FJ406448.1	S. coeruleorubidus ATCC 13740	0.99	97.89	CP023694.1
	S. odorifer NBRC 13365	0.98	99.37	NR_112382.1								
AT60	S. praecox CSSP720	0.99	100	NR_115437.1	S. pratensis ch24	1	99.81	JQ806242.1	S. fulvissimus DSM 40593	0.99	99.86	CP005080.1
	S. cyaneofuscatus CSSP436	0.99	99.93	NR_115383.1	K. papulosa NRRL B-16504	1	99.63	KJ996383.1	S. californicus FDAARGOS_1209	0.99	99.71	CP070242.1
	S. pratensis ch24	0.99	99.93	NR_125619.1	S. mutomycini AS 4.1747	1	98.7	EF055100.1	S. bacillaris ATCC 15855	0.99	99.35	CP029378.1
	S. anulatus NBRC 13369	0.99	99.93	NR_112527.1	S. atroolivaceus AS 4.1405	1	98.33	EF055078.1	S. nitrosporeus ATCC 12769	0.99	99.35	CP023702.1
	K. papulosa JCM 7250	0.99	99.49	NR_126320.1	S. olivoviridis NRRL B-3374	1	98.15	KJ996733.1				
	S. praecox NBRC 13073	0.99	99.93	NR_112358.1								
AT67	S. sanglieri NBRC 100784	0.97	99.36	NR_041417.1	S. atratus AS.4.1632	1	95.93	EF661770.1	S. nitrosporeus ATCC 12769	0.98	98.93	CP023702.1
	S. atratus NBRC 3897	0.97	99.29	NR_112503.1	S. cyaneofuscatus AS 4.1612	1	95.93	EF055088.1	S. californicus FDAARGOS_1209	0.98	98.86	CP070242.1
	S. atratus NRRL B-16927	0.97	99.29	NR_043490.1	S. bacillaris ATCC 15855	1	95.74	CP029378.1	S. fulvissimus DSM 40593	0.98	98.79	CP005080.1
	S. pulveraceus NBRC 3855	0.97	99.15	NR_041213.1	S. flavogriseus AS 4.1884	1	95.74	EF055115.1	S. bacillaris ATCC 15855	0.98	98.72	CP029378.1
	S. gelaticus NBRC 12866	0.97	99.01	NR_112308.1	S. griseobrunneus AS 4.1838	1	95.74	EF055108.1	S. lunaelactis MM109	0.98	98.64	CP026304.1
AT92	S. cavourensis NBRC 13026	1	100	NR_112345.1	S. aureoversilis NRRL B-3325	1	99.63	KJ996724.1	S. bacillaris ATCC 15855	1	99.42	CP029378.1
	S. cavourensis NRRL 2740	1	99.93	NR_043851.1	S. tendae NRRL B-2313	1	98.7	KT389053.1	S. californicus FDAARGOS_1209	1	99.28	CP070242.1
	K. albolonga NBRC 13465	1	99.86	NR_041144.1	S. plicatus NRRL 2428	1	98.52	KJ996182.1	S. fulvissimus DSM 40593	1	99.06	CP005080.1
	S. griseobrunneus NBRC 12775	1	99.86	NR_112577.1	S. desiacus NRRL B-1773	1	98.33	KJ996420.1	S. lunaelactis MM109	1	98.99	CP026304.1
	S. griseobrunneus CSSP424	1	99.71	NR_115448.1	S. rochei NRRL 3533	1	98.33	KJ996201.1	S. nitrosporeus ATCC 12769	1	98.92	CP023702.1
AT95	S. praecox CSSP720	0.97	100	NR_115437.1	K. papulosa NRRL B-16504	1	99.81	KJ996383.1	S. fulvissimus DSM 40593	0.98	99.85	CP005080.1
	S. cyaneofuscatus CSSP436	0.97	99.93	NR_115383.1	S. pratensis ch24	1	99.63	JQ806242.1	S. californicus FDAARGOS_1209	0.98	99.71	CP070242.1
	S. pratensis ch24	0.97	99.93	NR_125619.1	S. mutomycini AS 4.1747	1	98.52	EF055100.1	S. bacillaris ATCC 15855	0.98	99.35	CP029378.1
	S. anulatus NBRC 13369	0.97	99.93	NR_112527.1	S. atroolivaceus AS 4.1405	1	98.52	EF055078.1	S. nitrosporeus ATCC 12769	0.98	99.35	CP023702.1
	S. mutomycini NBRC 100999	1	99.85	NR_041421.1	S. olivoviridis NRRL B-3374	1	98.33	KJ996733.1				
	S. atroolivaceus NBRC 12741	1	98.63	NR_112255.1								
	S. praecox NBRC 13073	0.97	98.7	NR_112358.1								

AT98	S. sampsonii NBRC 13083	0.98	99.57	NR_112362.1	S. canescens AS 4.1681	1	100	FJ406440.1	S. koyangensis VK-A60T	0.98	99.29	CP031742.1
	S. coelicolor NBRC 12854	0.98	99.57	NR_112305.1	S. odorifer NBRC 13365	1	99.81	FJ406446.1	S. cadmiisoli ZFG47	0.98	98.43	CP030073.1
	S. limosus NBRC 12790	0.98	99.57	NR_112279.1	S. argenteolus AS 4.1693	1	99.81	EF055099.1	S. pluripotens MUSC 135	0.98	98.22	CP021080.1
	S. felleus NBRC 12766	0.98	99.57	NR_112266.1	S. vinaceus AS 4.1305	1	99.81	EF055073.1		0.98	98.15	AP023439.1
	S. canescens DSM 40001	0.98	99.5	NR_119346.1	S. coelicolor DSM 40233	1	99.63	FJ406448.1				
AT99	S. termitum NBRC 13087	0.96	98.88	NR_041112.1	S. omiyaensis NRRL B-1587	1	97.23	KJ996337.1	S. cinereoruber ATCC 19740	0.97	98.8	CP023693.1
	S. cinereoruber CSSP440	0.96	98.8	NR_043345.1	S. roseolus NRRL B-5424	1	97.04	KT389028.1	S. laurentii ATCC 31255	0.97	98.59	AP017424.1
	S. cinereoruber NBRC 12756	0.96	98.8	NR_112261.1	S. nashvillensis NRRL B-2606	1	96.85	KT388976.1	S. venezuelae ATCC 10712	0.97	97.96	CP029197.1
	S. viridobrunneus NBRC 15902	0.95	99.07	NR_112481.1	S. bikiniensis NRRL B-1049	1	96.3	KJ996209.1	S. dengpaensis XZHG99	0.97	97.68	CP026652.1
	S. viridobrunneus LMG 20317	0.96	98.6	NR_042308.1	S. cinereoruber ATCC 19740	1	96.12	CP023693.1				
AT120	S. praecox CSSP720	0.99	99.93	NR_115437.1	S. pratensis ch24	1	99.81	JQ806242.1	S. fulvissimus DSM 40593	0.99	99.71	CP005080.1
	S. cyaneofuscatus CSSP436	0.99	99.86	NR_115383.1	K. papulosa NRRL B-16504	1	99.63	KJ996383.1	S. californicus FDAARGOS_1209	0.99	99.57	CP070242.1
	S. pratensis ch24	0.99	99.86	NR_125619.1	S. mutomycini AS 4.1747	1	98.7	EF055100.1	S. nitrosporeus ATCC 12769	0.99	99.21	CP023702.1
	S. anulatus NBRC 13369	0.99	99.86	NR_112527.1	S. atroolivaceus AS 4.1405	1	98.33	EF055078.1	S. bacillaris ATCC 15855	0.99	99.21	CP029378.1
	S. praecox NBRC 13073	0.99	99.86	NR_112358.1	S. olivoviridis NRRL B-3374	1	98.15	KJ996733.1				

References

1. Heuer H, Krsek M, Baker P, Smalla K, Wellington EM. 1997. Analysis of actinomycete communities by specific amplification of genes encoding 16S rRNA and gel-electrophoretic separation in denaturing gradients. Appl Environ Microbiol 63:3233-3241.

2. Sheffield VC, Cox DR, Lerman LS, Myers RM. 1989. Attachment of a 40-base-pair G + C-rich sequence (GC-clamp) to genomic DNA fragments by the polymerase chain reaction results in improved detection of single-base changes. Proc Natl Acad Sci U S A 86:232-236.
